# Histone Deacetylase Inhibitors Downregulate Checkpoint Kinase 1 Expression to Induce Cell Death in Non-Small Cell Lung Cancer Cells

**DOI:** 10.1371/journal.pone.0014335

**Published:** 2010-12-14

**Authors:** William Brazelle, Jenny M. Kreahling, Jennifer Gemmer, Yihong Ma, W. Douglas Cress, Eric Haura, Soner Altiok

**Affiliations:** Thoracic Oncology and Experimental Therapeutics Programs, H. Lee Moffitt Cancer Center and Research Institute, Tampa, Florida, United States of America; University of Giessen Lung Center, Germany

## Abstract

**Background:**

Histone deacetylase inhibitors (HDACis) are promising anticancer drugs; however, the molecular mechanisms leading to HDACi-induced cell death have not been well understood and no clear mechanism of resistance has been elucidated to explain limited efficacy of HDACis in clinical trials.

**Methods and Findings:**

Here, we show that protein levels of checkpoint kinase 1 (Chk1), which has a major role in G_2_ cell cycle checkpoint regulation, was markedly reduced at the protein and transcriptional levels in lung cancer cells treated with pan-and selective HDACis LBH589, scriptaid, valproic acid, apicidin, and MS-275. In HDACi treated cells Chk1 function was impaired as determined by decreased inhibitory phosphorylation of cdc25c and its downstream target cdc2 and increased expression of cdc25A and phosphorylated histone H3, a marker of mitotic entry. In time course experiments, Chk1 downregulation occurred after HDACi treatment, preceding apoptosis. Ectopic expression of Chk1 overcame HDACi-induced cell death, and pretreating cells with the cdc2 inhibitor purvalanol A blocked entry into mitosis and prevented cell death by HDACis. Finally, pharmacological inhibition of Chk1 showed strong synergistic effect with LBH589 in lung cancer cells.

**Conclusions:**

These results define a pathway through which Chk1 inhibition can mediate HDACi-induced mitotic entry and cell death and suggest that Chk1 could be an early pharmacodynamic marker to assess HDACi efficacy in clinical samples.

## Introduction

Histone deacetylase inhibitors (HDACis) represent a promising new class of compounds for the treatment of cancer [1]. Some HDACis show broad activity against multiple HDAC classes (scriptaid, LBH589), whereas others are class-or isotype selective (MS-275) [1], [2].

The precise mechanisms by which HDACis exert their cytotoxic effects are unknown; however, the antitumor effects of these drugs are thought to result from hyperacetylation of histones, demethylation of genomic DNA, and activation of genes that inhibit proliferation and induce apoptosis [1]. In addition to their epigenetic effects, HDACis have also been shown to have significant post-translational effects on non-histone proteins, including transcription factors p53, heat-shock protein 90 (HSP90), and α-tubulin [3]. Besides direct anti-tumorigenic effects, suppression of angiogenesis by HDACis might also have an impact on tumor growth inhibition [4].

An essential regulatory step for the G_2_/M cell cycle transition in eukaryotes is activation of the cdc2-cyclin B complex [5]. The proper regulation of cdc2 requires an activating phosphorylation on threonine-161 and inhibitory phosphorylations on threonine-14 and tyrosine-15 (Tyr15) [6]. The inhibitory phosphorylation on Tyr15 maintains the cdc2-cyclin B complex in an inactive state if there is incompletely replicated DNA or damaged DNA in the cell [7], [8]. Cdc2 activation through removal of its inhibitory phosphorylation by cdc25 phosphatases allows cells to enter the mitotic phase of the cell cycle [9]. Chk1 is a critical component of DNA replication, intra-S phase, G_2_/M phase, and mitotic spindle-assembly checkpoints [10]. In response to a variety of genotoxic stressors, Chk1 becomes activated by upstream kinases such as ATM and ATR, leading to increased proteosomal degradation of the phosphatase cdc25A and inhibition of cdc25C through serine-216 (Ser216) phosphorylation, collectively causing inactivation of cdc2 and consequently G_2_/M arrest [10]–[14].

Furthermore, combining HDACis with regulators of G2 checkpoint could improve efficacy and aid in overcoming resistance. In fact, direct pharmacologic inhibition or siRNA knockdown of Chk1 has been shown to cause checkpoint abrogation, cytokinetic regression, and multinucleation, as well as chromosome missegregation and chromosomal instability [15]. Therefore, Chk1 inhibitors, which effectively abrogate the S and G_2_ checkpoints, are being investigated in clinical trials either alone or in combination with cytotoxic agents [16]–[18] and could be effectively combined with HDACi to enhance cytotoxic effects.

Here, we demonstrate that HDACi treatment downregulates Chk1 protein expression, which in turn leads to unscheduled cdc2 activation, mitotic entry, and cell death in human lung cancer cells. The results of this study demonstrate that Chk1 downregulation and abrogation of G_2_ checkpoint are important regulatory steps in sensitivity and resistance to the cytotoxic effect of HDACi treatment in non-small cell lung cancer (NSCLC) cells. Our data suggest that Chk1 might be an early pharmacodynamic marker to predict and assess the efficacy of HDACis and Chk1 inhibitors may enhance cytotoxic effects of HDACis in clinical studies.

## Results

### Treatment of NSCLC cells with HDACis causes G_2_/M cell cycle arrest and cell death

**Figure 1:**
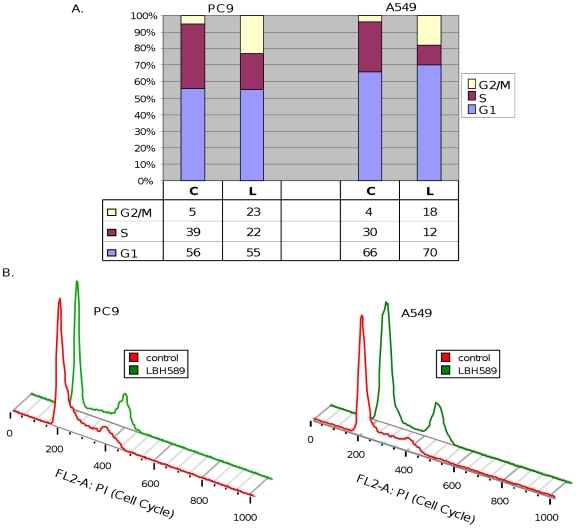
Effect of LBH589 on cell cycle distribution of A549 and PC9 cells. *A*, The asynchronously growing cells were incubated in medium with 5% FCS for 24 hours in the absence (control, C) or presence (L) of LBH589. The percentage of cells in different phases of the cell cycle was determined by flow cytometric analysis. *B*, Histogram representation of cell cycle data determined by flow cytometric analysis.

Previous studies have demonstrated that a pan-HDACi LBH589 (IC_50_ ranging between approximately 9 and 54 nmol/L) causes growth arrest and cell death in NSCLC cells [19]. To analyze the molecular mechanisms by which HDACis regulate cell cycle progression and cell death, asynchronously growing A549 (EGFR wild type, K-Ras mutant, and p53 wild type) and PC9 (EGFR mutant, K-ras wild type, and p53 mutant) [20]–[22] cells were treated with LBH589 (40 nM) for 24 hours and collected for flow cytometric analysis. Figure 1 shows that in PC9 and A549 cells LBH589 treatment produced a noticeable increase in the cells in the G_2_/M phase suggestive of a G_2_/M blockade and a significant reduction in S-phase cells. These results are in agreement with the previous reports that HDACis lead to G_2_/M arrest and apoptotic cell death in NSCLC cells [19].

**Figure 2:**
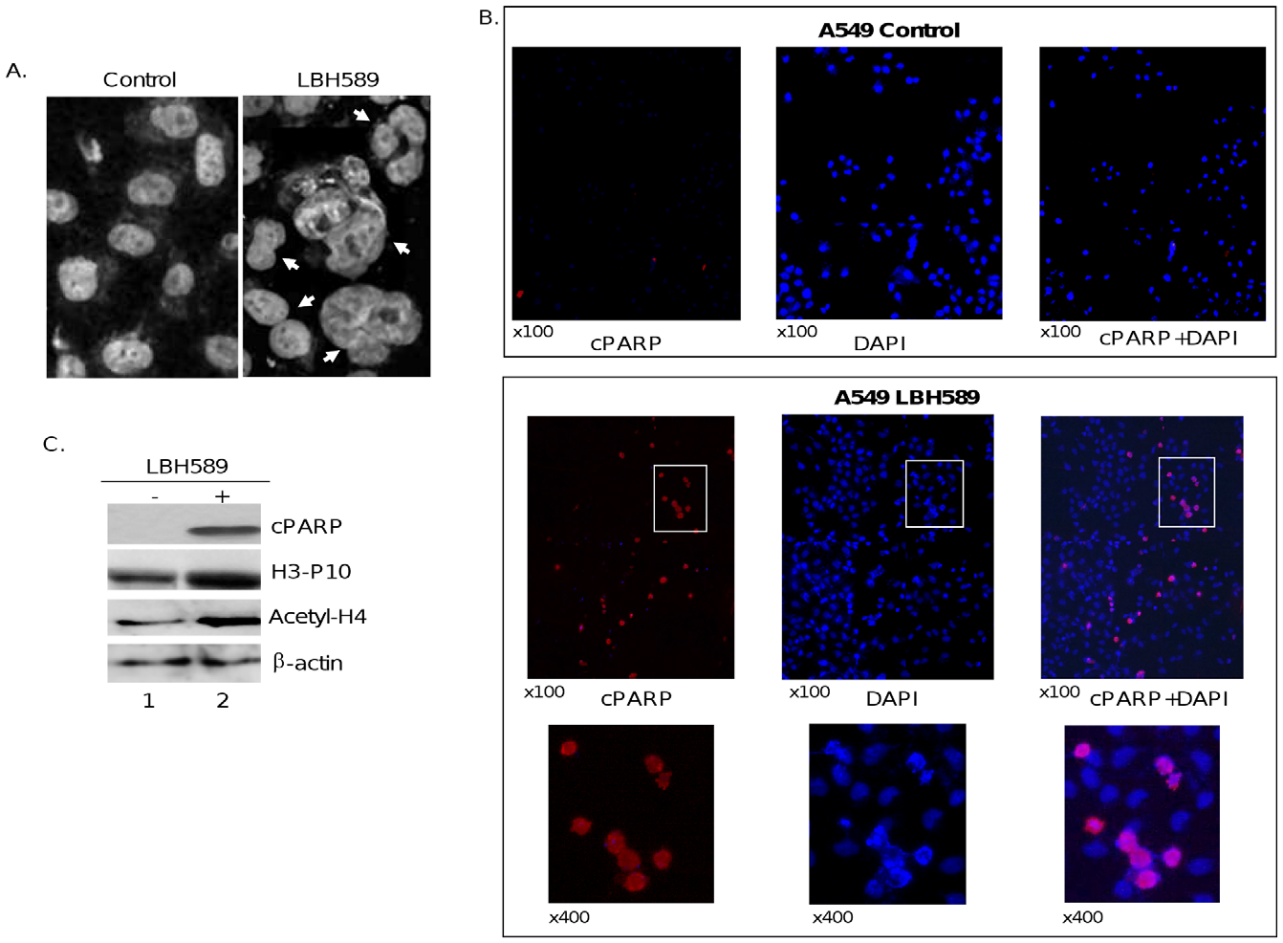
LBH589 treatment leads to mitotic abnormalities and cytokinesis failures. A549 cells were treated with vehicle (control) or 40 nM LBH589 for 24 hours. *A*, arrows show bi- or multinucleated cells with impaired cytokinesis in LBH589-treated NSCLC cells. *B*, cells were fixed and stained with DAPI or cleaved poly (ADP-ribose) polymerase (cPARP) antibody (×100 or ×400 magnification). *C*, Western blot analysis demonstrating that HDAC inhibition by LBH589 causes histone H3 phosphorylation (H3-P10), histone H4 acetylation (Acety-H4), and PARP cleavage (cPARP) in A549 cells treated with LBH589 for 24 hours. β-actin was used as loading control.

As illustrated in Figure 2A, microscopic examination of cells treated with LBH589 produced a range of different nuclear morphologies, from binucleation to multinucleation. Taken together, these data show that LBH589 treatment causes cell death associated with abnormal mitosis and failed cytokinesis, which is suggestive of mitotic catastrophe, a type of cell death that occurs during mitosis and has previously been described in cells treated with the HDACi trichostatin A [23].

**Figure 3:**
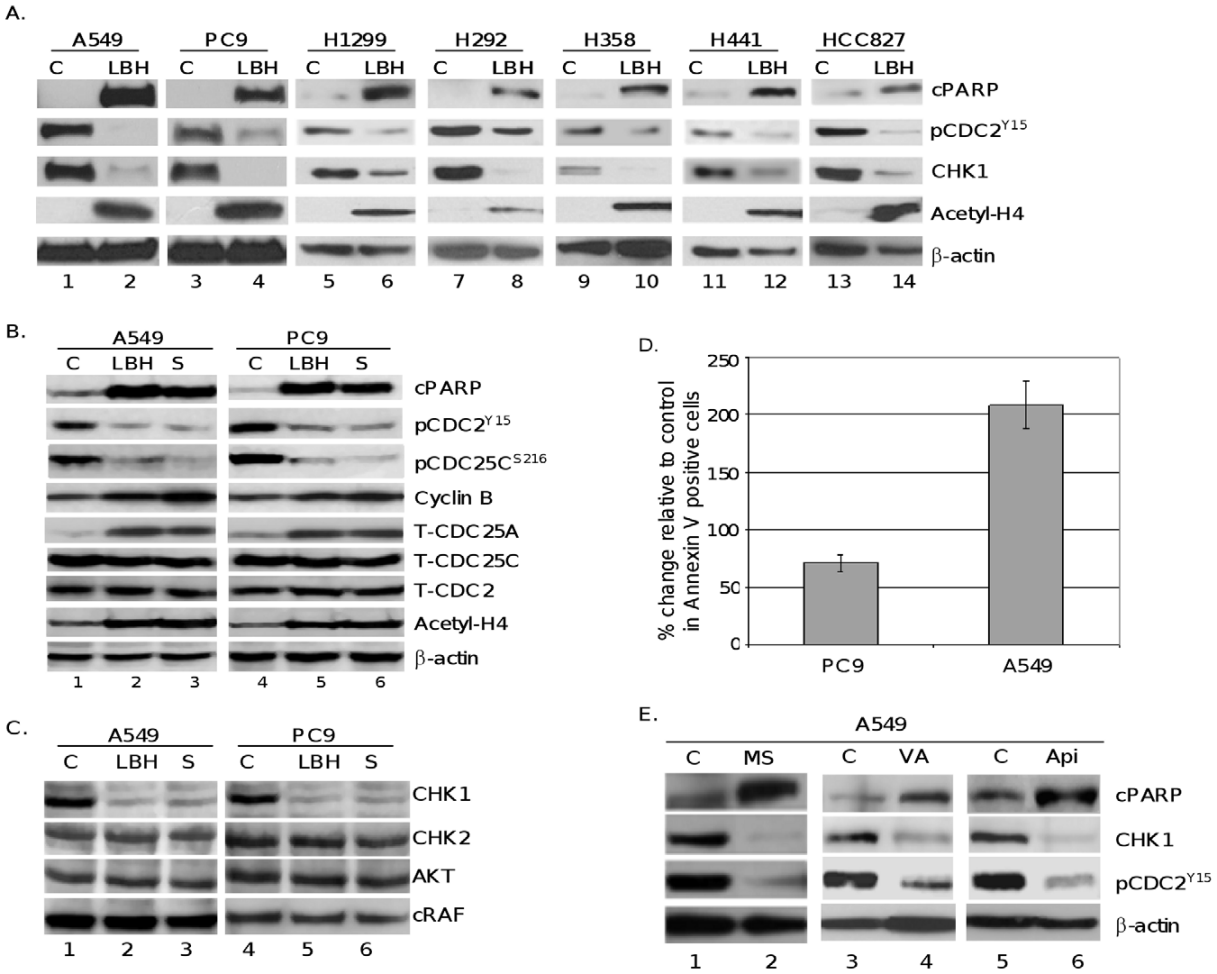
HDACi treatment specifically inhibits CHK1 expression and upregulates its downstream signaling proteins CDC25A, CDC25C, and CDC2, involved in G_2_ cell cycle checkpoint control. *A*, A549, PC9, H1299, H292, H358, H441 and HCC827 cells were cultured in the presence of vehicle (C), or LBH589 (LBH) 40 nM for 24 hours and expression levels of cPARP, phosphorylation of CDC2 (pCDC2 ^Y15^), CHK1, and acetylated histone H4 (acetyl-H4) were determined by Western blot and quantitated using AlphaEase software. β-actin was used as loading control. *B*, PC9 and A549 cells were cultured in the presence of vehicle (C), or LBH589 (LBH) 40 nM, or scriptaid (S) 1 µM for 24 hours and expression levels of cPARP, tyrosine-15 phosphorylation of CDC2 (pCDC2 ^Y15^), serine-216 phosphorylation of CDC25C (pCDC25c ^S216^), CDC25A (T-CDC25A), CDC25C (T-CDC25C) and CDC2 (T-CDC2), acetylated histone H4 (acetyl-H4), and cyclin B1 were determined by Western blot analysis. β-actin was used as loading control. *C*, drug-mediated changes in the expression of CHK1, CHK2, AKT, and c-RAF were determined by Western blot analysis. β-actin was used as loading control. *D*, PC9 or A549 cells were treated with or without 40 nM LBH589 and analyzed for annexin positive cells using the BD Annexin V-FITC/7-AAD Flow Cytometry kit. *E*, A549 cells were treated with MS-275 (MS), (500 nM), valproic acid (VA) (1 Mm), or apicidin (Api) (400 nM) for 24 h. Expression levels of cPARP, CHK1, pCDC2 ^Y15^, and β-actin were determined by Western blot analysis. All experiments were repeated at least three times.

Since cells in the G_2_- and M-phase could not be discriminated based on their differences in DNA content by flow cytometric analysis, we tested whether cells arrested in G_2_/M after HDACi treatment enter mitotic phase. For this purpose, we first performed immunofluorescence staining using the antibody against cleaved poly(ADP-ribose) polymerase (cPARP) and showed that LBH589 treatment causes apoptotic cell death in 78% of binucleated mitotic cells (Figure 2B). Next, in Western blot analysis we tested whether HDAC inhibition enhances histone H3 phosphorylation at serine-10, which takes place at the onset of mitosis. Figure 2C illustrates that LBH589 treatment caused histone H3 phosphorylation accompanied by increased histone acetylation and PARP cleavage, indicating that HDACi treatment causes NSCLC cells to enter mitosis and undergo cell death. To determine whether cell growth and viability changes observed after LBH589 treatment are a generalized effect of HDAC inhibition, we also used another pan-HDACi, scriptaid (1 µM), which produced biochemical changes indistinguishable from those presented above using LBH589 (Figure 3).

### HDAC inhibition results in activation of cdc25 and cdc2 proteins that are required for mitotic entry

Activation of the cdc2 protein kinase by the cdc25 protein phosphatases is a pivotal regulatory step for the G_2_/M transition in eukaryotes [9]. The dephosphorylation of cdc25C on Ser216 increases its activity during M-phase transition, leading to activation of the cdc2 kinase [10]. To investigate whether HDACi-induced mitotic entry and cell death are mediated by activation of the cdc25/cdc2 pathway, PC9, A549, H1299, H292, H358, H441 and HCC827 cells were treated with or without LBH589, and Western blot analysis was performed. Figure 3A shows HDACi treatment resulted in increased levels of PARP cleavage, a decrease in the inhibitory phosphorylation on cdc2 and 45–94% decrease (based on quantitative Western blot analysis) in total Chk1 across the 7 different NSCLC cell lines, with A549s showing the most significant decrease in Chk1 protein. These changes are all associated with an increase in histone acetylation. Further investigation of A549 and PC9 cells treated with pan HDACis (Figure 3B), LBH589 and scriptaid, showed activation of cdc25C and cdc2, as assessed by a decrease in their inhibitory phosphorylation on Ser216 and Tyr15, respectively, which were accompanied by increased histone H4 acetylation. No decrease was observed in the expression levels of total cdc25C and cdc2 proteins (Figure 3B). These results indicate that HDACi-induced mitotic entry is mediated by increased cdc25/cdc2 activity. Furthermore, HDACi treatment also increased the expression level of cdc25A, which is targeted by Chk1 for rapid degradation (Figure 3B). These data show that, in HDACi-treated cells, the biologic function of Chk1 was inhibited.

Besides phosphorylation at the Tyr15 position, another major mechanism regulating cdc2 activity and progression into mitotic phase of the cell cycle is its complex formation with cyclin B, a required cofactor for the cdc2 kinase activity. Thus, we next determined whether HDACi treatment affects cyclin B expression levels. As shown in Figure 3B, NSCLC cells treated with LBH589 or scriptaid displayed higher overall levels of cyclin B1 expression. Taken together with the decreased Tyr15 phosphorylation of cdc2, this finding implies a high cdc2-cyclin B activity in HDACi-treated cells, providing further support that cells treated with HDACis are capable to enter mitosis.

### Inhibition of HDAC activity correlates with decreased Chk1 expression in NSCLC cells

The order and fidelity of the cell cycle are linked to the integrity of the Chk1 and cdc25 phosphatase pathways. Maintenance of the Chk1/cdc25 pathway is essential for cells to progress normally through an unperturbed cell division cycle and for cells to arrest in response to checkpoint activation [10].

To determine the role of Chk1 in HDACi-induced activation of cdc25C and cdc2, we analyzed changes in the levels of Chk1 protein in cells treated with HDACis. Immunoblot analysis revealed that treatment with LBH589 or scriptaid causes a significant decrease in the expression of Chk1 protein (Figure 3C). This inhibition appears to be specific since no change was observed in the expression of Chk2 protein, another regulator of DNA-damage checkpoint signaling pathway [10], [11].

HDACis, including LBH589, have been reported to functionally inactivate HSP90 through protein acetylation, leading to depletion of the client proteins such as AKT and c-RAF [22]. Interestingly, Chk1 protein has also been reported to be an HSP90 client [24], [25], suggesting that inactivation of HSP90 by HDACis might be responsible for degradation of Chk1 observed in our experiments. To test this possibility, we analyzed whether Chk1 downregulation coincides with decreased expression of AKT and c-RAF in extracts prepared from cells treated with LBH589 or scriptaid. As illustrated in Figure 3C, no changes were observed in the expression levels of AKT or c-RAF proteins under the conditions where LBH589 and scriptaid caused Chk1 downregulation.

To further demonstrate the cytotoxic effects of HDACi treatment on NSCLC cells we measured apoptotic cell death using Annexin V detection. As shown in Figure 3D, PC9 and A549 cells showed a measured increase in the amount of annexin positive cells indicating that treatment with HDACi results in significant increase in cells undergoing apoptosis.

To determine whether selective HDACis also cause Chk1 inhibition, A549 and PC9 cells were treated with MS-275 that selectively inhibits HDAC1, and to lesser extent HDAC3. As illustrated in Figure 3E, MS-275 (0.5 µM) produced changes in the expression of Chk1, phosphorylated cdc2 (Tyr15), and cPARP similar to results observed with LBH589 and scriptaid. Two other class I HDACis valproic acid (1 mM) and apicidin (400 nM) also produced identical results (Figure 3E). These data demonstrate that HDACi-mediated Chk1 downregulation is not drug dependent and class I HDACs are likely mediators of the HDACi effect on Chk1 downregulation in NSCLC cells.

### Chk1 abrogation precedes initiation of apoptosis by LBH589

**Figure 4:**
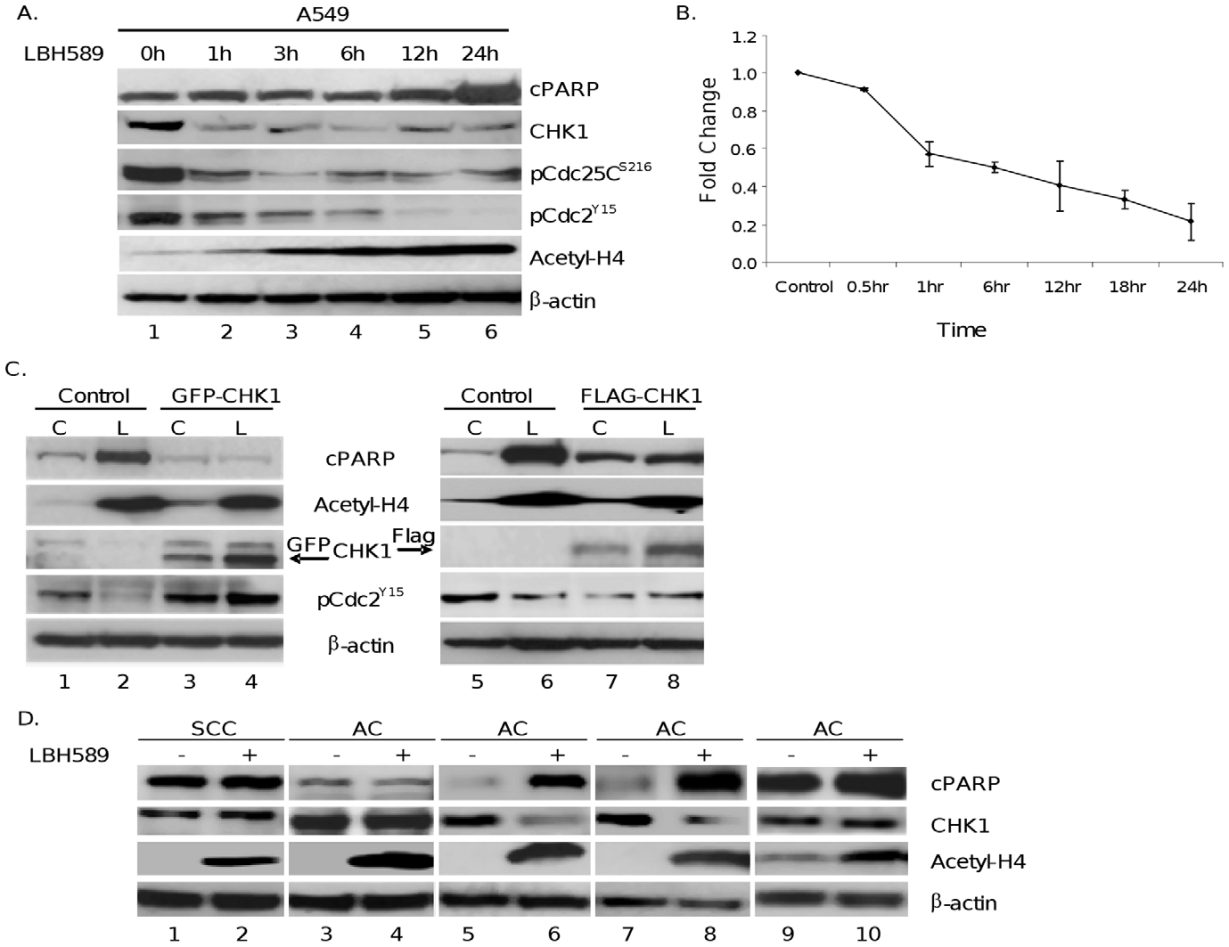
*A*, HDACi-mediated decrease in CHK1 precedes apoptosis. A549 cells were untreated or treated with LBH589 (40 nM) for various time points. Cell lysates were prepared, and protein expression levels of cPARP, CHK1, Tyr15 phosphorylation of pCDC2 (pCDC2 ^Y15^), Ser216 phosphorylation of CDC25C (pCDC25 ^S216^), acetyl-H4, and cyclin B1 were determined. *B*, Quantitative Chk1 mRNA expression analysis. Total RNA was prepared from A549 cells after 24 hours of treatment with 40 nM LBH589 or vehicle. mRNA expression levels were quantified using Real-time PCR analysis. All results were normalized to GAPDH mRNA levels, and the mean and standard deviations values from four independent experiments are shown. *C*, Ectopic expression of Chk1 reverses HDACi-induced apoptosis but not histone acetylation. A549 cells were transiently transfected with an empty vector (control) or GFP- or FLAG-tagged (GFP-CHK1 or FLAG-CHK1) Chk1 expression plasmid. Forty-eight hours after transfection, cells were cultured without (control, C) or with LBH589 (L) (40 nM) for an additional 24 hour before harvesting for Western blot analysis. Treatment-induced changes in cPARP, acetyl-H4, phospho-CDC2 ^Y15^, and ectopically expressed GFP-CHK1 or FLAG-CHK1 proteins were determined by Western blot analysis. β-actin expression was used as loading control. Experiments were repeated 3 times, and a representative experiment is shown. The arrows show the position of the GFP-CHK1 and FLAG-CHK1 proteins. *D*, in primary NSCLC patient samples, Chk1 protein downregulation correlates with increased cPARP *ex vivo*. Tumor samples were collected with a 23-gauge needle from patient-derived tumors, and cells were treated in duplicate with vehicle (control) or LBH589 (40 nM) for 18 hours. Following treatment, adherent and non-adherent cells were pooled, cell extracts were prepared, and expression levels of cPARP, Chk1, and acetyl-H3 were analyzed by Western blot. β-actin expression was used as loading control. SCC: squamous cell carcinoma; AC: adenocarcinoma.

Chk1 has been reported to be subject to caspase-mediated degradation in cells undergoing apoptotic cell death [26]. Consequently, we analyzed in time course experiments whether downregulation of Chk1 protein observed in HDACi-treated cells is the cause or the consequence of apoptotic cell death. As shown in Figure 4A, treatment of A549 cells with LBH589 led to a 70% decrease in Chk1 protein within 1 hour of treatment, based on quantitation of western blots, which was accompanied by decreased phospho-cdc25C (Ser216) and phospho-cdc2 (Tyr15) levels consistent with functional inactivation of Chk1 activity. Of importance, these changes occurred before detectable caspase activation, as evidenced by PARP cleavage that was first observed at 12 hours of treatment. A similar time course profile was observed in PC9 cells treated with LBH589 or scriptaid (data not shown).

Collectively, these results support a model where inhibition of Chk1 expression by HDACis leads to cell death through unscheduled activation of the mitosis-promoting kinase cdc2 in NSCLC cells.

### HDACi treatment leads to the transcriptional downregulation of Chk1

To determine whether HDACi treatment regulates Chk1 expression at the transcriptional level in NSCLC cells, we performed real-time PCR. As illustrated in Figure 4B, quantitative RNA analysis revealed more than a 50% reduction in Chk1 mRNA levels in A549 cells 24 hours after LBH589 (40 nM) treatment. This finding demonstrates that HDACi treatment leads to transcriptional repression of Chk1 in NSCLC cells.

### Ectopic expression of Chk1 overcomes LBH589-induced apoptosis

To determine the importance of Chk1 downregulation in HDACi-induced apoptotic cell death, we aimed to determine whether ectopic expression of Chk1 can overcome cell death triggered by LBH589. Therefore, A549 cells were transiently transfected with a Chk1 plasmid encoding a GFP-tagged Chk1 fusion protein. As shown in Figure 4C, transient expression of ectopic Chk1 protein suppressed LBH589-induced apoptosis, as detected by PARP protein cleavage. Additionally, in cells expressing ectopic Chk1, LBH589 treatment failed to activate cdc2, as assessed by Tyr15 dephosphorylation (Figure 4C), while no changes were observed in HDACi-induced histone acetylation. Similar data were obtained with a Flag-tagged Chk1 expression plasmid in NSCLC cells (Figure 4C). These results indicate that Chk1 downregulation plays an essential role in HDACi-mediated cell death.

### Chk1 downregulation correlates with apoptotic cell death in primary NSCLC cells treated with LBH589 *ex vivo*

To verify the downregulation of Chk1 in clinically relevant tumor samples, tumor cells collected from NSCLC patient tumors were treated ex vivo with LBH589. Total proteins were extracted, and drug-mediated changes in the expression of Chk1, histone acetylation, and PARP cleavage were analyzed by Western blot. As illustrated in Figure 4D, ex vivo treatment of cells with LBH589 (40 nM) caused increased histone H4 acetylation in all tumor samples, whereas, LBH589 treatment increased PARP cleavage in only 2 of 5 samples, which closely correlates with Chk1 downregulation. These results support the findings presented above with NSCLC cell lines and suggest that decreased Chk1 and phospho-cdc2 (Tyr15) levels may be sensitive pharmacodynamic markers for HDACi efficacy in patient tumor samples to predict and assess patient response to HDACi treatment in clinical studies.

### HDACi-mediated cell death requires mitotic entry

**Figure 5:**
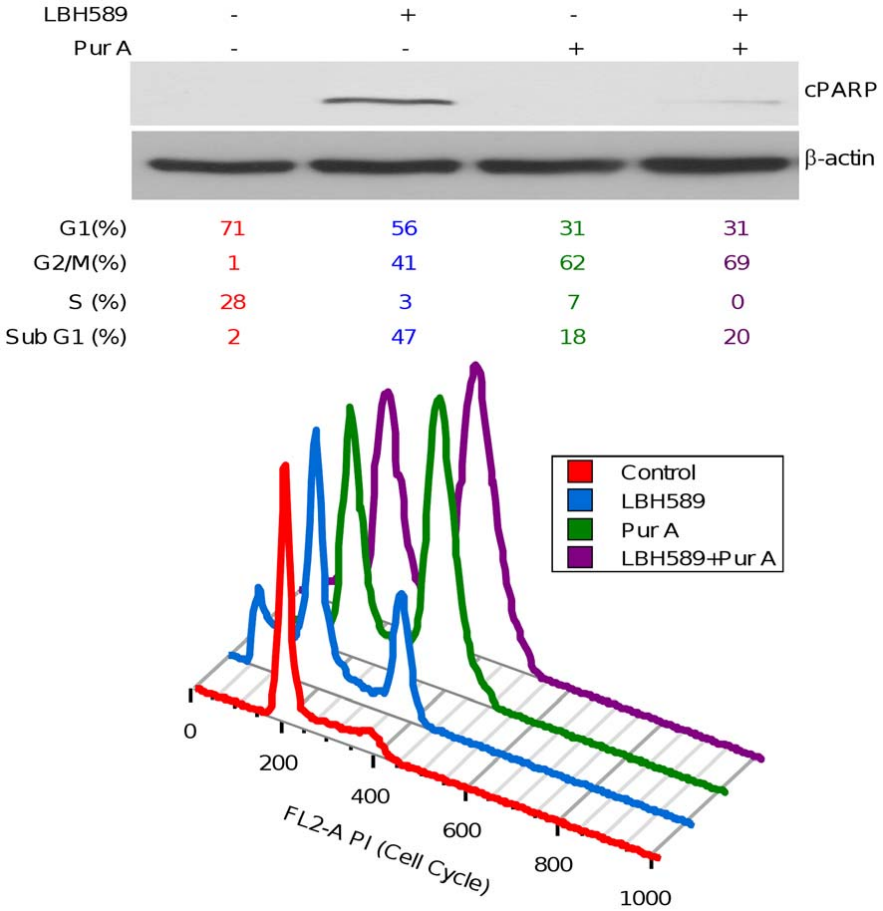
Purvalanol A (Pur A) pretreatment diminishes the cytotoxic effect of LBH589 in A549 cells. Cells treated with LBH589 40 nM with or without Pur A (10 µM) pretreatment were analyzed by flow cytometry for cell cycle distribution or by Western blot to determine drug-mediated changes in cPARP. β-actin was used as a loading control.

The data presented above support a model where failure of Chk1-mediated checkpoint activation plays an essential role in HDACi-mediated cell death. Thus, we hypothesize that blockade of mitotic entry would diminish the sensitivity of cancer cells to the cytotoxic effects of HDACi treatment. To test this hypothesis, A549 cells were pretreated with a potent cdc2 inhibitor, purvalanol A [27], to block entry into mitosis. Flow cytometric analysis illustrates that 40 nM LBH589 for 24 hours caused a significant increase of cells in G_2_/M and an approximately 20-fold increase of the sub-G_1_ peak (hypodiploid cells) after 24 hours of treatment, consistent with increased cell death. The increase in the sub-G_1_ population after LBH589 treatment, however, was significantly reduced by pretreatment of cells with purvalanol A (Figure 5), indicating that mitotic blockade causes resistance to the cytotoxic effects of LBH589. To confirm this observation, we performed Western blot analysis with extracts prepared from A549 cells exposed to LBH589 with or without purvalanol A pretreatment. Figure 5 illustrates that purvalanol A pretreatment inhibited LBH589-induced apoptotic cell death, as measured by cPARP, providing further support that HDACi-induced cell death largely requires entry into mitosis.

### HDAC and Chk1 inhibitors show a synergistic effect in NSCLC cells

**Figure 6:**
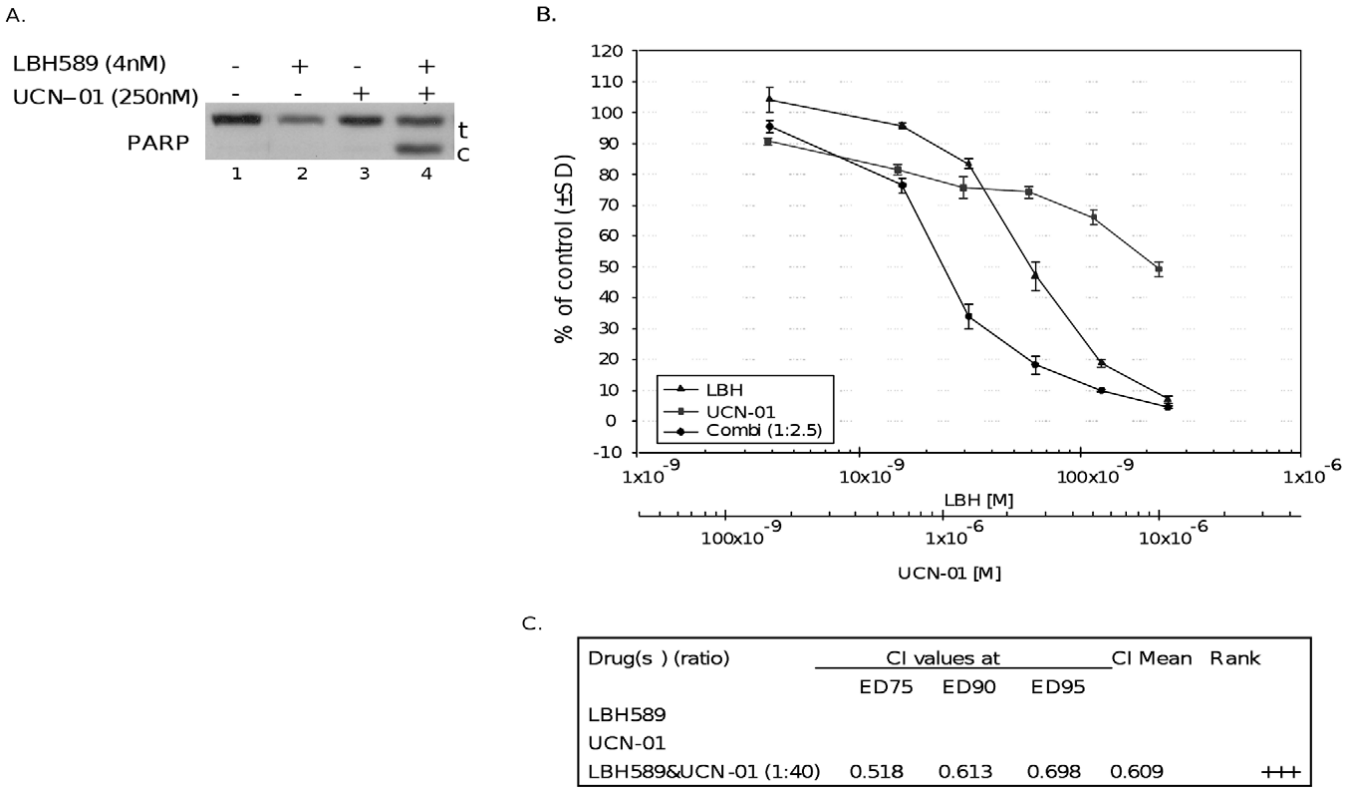
LBH589 and Chk1 inhibitor treatment shows a synergistic effect in NSCLC cells. *A*, A549 cells were treated with LBH589 40 nM and/or a UCN-01 (250 nM) for 24 hours, cell extracts were prepared, and Western blot analysis was performed with PARP (t: total, c: cleaved). Experiments were repeated at least 3 times, and a representative experiment is shown. *B*, A549 cells were treated with LBH589 and UCN-01 either alone or in combination at a constant ratio (1∶40) for 72 hours. Drug concentrations are indicated on the horizontal axis and plotted against cell viability of control wells, which was arbitrarily set at 100% viability for each experiment. Error bars represent ± SD of 4 replicate wells. *C*, combined effects of LBH589 and Chk1 inhibitor UCN-01 were quantified with the Chou and Talalay combination index (CI) method (40). The CI used for drug combination analyses was determined by the isobologram equation (see text). Ranking symbols (+/−) indicate average calculated Chou and Talalay combination index (CI) range (+++, strong synergism).

Our results showed that HDACi treatment targets Chk1 to induce inappropriate mitotic entry and thereby exerts its cytotoxic effects on tumor cells. The diminished sensitivity of cells to HDACi treatment observed in A549 cells ectopically expressing Chk1 (Figure 4C) suggests that activation of Chk1 and its downstream signaling pathway, which control the G_2_ check point, may cause resistance to HDACi therapy. Thus, it is plausible that Chk1 inhibitors may potentiate cytotoxic effects of HDACis in tumor cells that are resistant to HDACis. To test this possibility, A549 cells were treated with LBH589 and a Chk1 inhibitor (UCN-01) for 24 hours. Figure 6A shows that, at low concentrations, neither LBH589 (4 nM) nor UCN-01 (250 nM) alone can cause PARP cleavage in A549 cells, whereas the combination of these two drugs drastically increased PARP protein cleavage, suggesting a potential synergy. To corroborate the synergistic interaction between HDAC and Chk1 inhibitors, we next performed median dose effect analysis. For this purpose, A549 cells were exposed to increasing concentrations of LBH589, UCN-01 or the combination of these drugs in a fixed ratio. Cell viability was assessed using CT-Blue assay, and the effect was analyzed using median effect analysis. As shown in Figures 6B and 6C, exposure of cells to the combination of LBH589 and Chk1 inhibitors exerted a strong synergistic effect. Using these same methods, a synergistic interaction was also seen between LBH589 and a novel Chk1 inhibitor AZD7762 [28] in NSCLC cells that resulted in a CI value of 0.76 indicative of a moderately synergistic effect (data not shown).

The data presented above suggest that drugs inhibiting the activity of proteins that negatively regulate the function of cdc2-cyclin B complex may enhance the G_2_ checkpoint abrogation and cytotoxic effects of HDACis in tumor cells.

## Discussion

Previous studies have shown that HDACi-induced cell death is associated with aberrant mitosis and disruption of the cell cycle checkpoints [29]–[34]. However, the molecular mechanisms by which HDACi treatment leads to mitotic cell death is not well understood. Here, we demonstrated that HDACi treatment of human NSCLC cells leads to downregulation of total Chk1 protein levels. We showed that this downregulation is biologically relevant by demonstrating a concomitant decrease in the levels of the inhibitory phosphorylation of key cell-cycle regulatory proteins cdc25c and cdc2. The decreased levels of total Chk1 protein preceded induction of apoptosis and histone acetylation, demonstrating that Chk1 downregulation is an early event in HDACi mode of action and that it plays a pivotal role in drug-mediated cell death.

Chk1 expression has been shown to fluctuate during the cell cycle [35]. However, since Chk1 is mainly expressed at the S to M phase of the cell cycle at both the RNA and protein levels [35], it is unlikely that HDACi-mediated inhibition of Chk1 is a consequence of growth arrest in G_2_/M phase. RT-PCT experiments demonstrated that transcriptional inhibition of Chk1 plays an important role in HDACi-mediated regulation of Chk1 protein. The mechanism by which HDACis regulate the transcriptional expression of Chk1 is unknown and remains to be elucidated.

Treatment of tumor cells with pan HDACis has been reported to cause acetylation of Hsp90 through inhibition of HDAC6 and depletion of several Hsp90 client proteins including AKT and c-RAF [22], [36], [37]. Our results showed that under the conditions where LBH589 induced Chk1 downregulation, no inhibition was observed in the expression levels of AKT and c-RAF. Furthermore, selective HDAC inhibitors valproic acid, apicidin and MS-275 with no known inhibitory effects on HDAC6 were able to inhibit Chk1 expression. Taken together, these results suggest that inhibition of Chk1 expression in HDACi-treated cells is likely not mediated by inactivation of HSP90 protein, but is rather due to repression of CHK1 expression.

One of the major challenges in the development of HDACis is the definition of the clinically relevant endpoints to assess drug efficacy in patient tumors at the early stage of treatment to predict clinical outcome. In the present study, we showed that downregulation of total Chk1 protein levels correlates much more strongly with induction of cytotoxic effect rather than with histone hyperacetylation in both cell lines and in primary NSCLC cells obtained from patient tumors ex vivo. Together, these findings suggest that downregulation of Chk1 and Tyr15 phosphorylation of cdc2 levels may be sensitive and clinically relevant pharmacodynamic markers of efficacy of HDACis.

Despite their promise as an emerging cancer therapy, in clinical trials, the effectiveness of HDACis has been limited, and no clear mechanism of resistance has been elucidated [1]. Our present results provide a possible insight into the mechanism of HDACi action and resistance. We showed that ectopic expression of Chk1 and prevention of mitotic entry by a cdc2 inhibitor purvalanol A diminished LBH589-induced cell death. These findings suggest that the level of Chk1 expression or activation status of enzymes regulating the M-phase entry, such as cdc25 and cdc2-cyclin B complex, may play an important role in determining sensitivity or resistance of tumor cells to HDACis. The data presented here suggest a model where mitotic entry is a required step for HDACi-mediated cell death. Additionally, cellular mechanisms that block Chk1 downregulation and/or bypass Chk1 and directly act on G_2_ checkpoint control to prevent cells from entering mitotic phase would cause resistance to HDACis. For example, Chk1 has been shown to be highly expressed in NSCLC tumors [38], which may confer resistance to HDACi-induced mitotic entry and cell death. Thus, in tumors with increased Chk1 activity, rational combinations of HDACis with drugs that simultaneously inhibit Chk1 and its downstream signaling pathways controlling G_2_/M cell cycle checkpoint may augment the cytotoxic effects of HDACis.

HDACis have been shown to synergize with DNA damaging agents [1]. However, the molecular mechanisms of this synergy have not well been understood. Since Chk1 is crucial for the G_2_ checkpoint activation and survival after treatment with DNA damaging agents, we speculate that inhibition of Chk1 might provide a molecular explanation for the enhancement of tumor sensitivity to chemotherapeutic agents by HDACis. Further studies are required to elucidate the precise role of Chk1 in the synergistic interaction of HDACis with the cytotoxic therapies to enhance their therapeutic efficacy in cancer treatment.

Here, we provide evidence that HDAC and Chk1 inhibitors show strong synergistic interaction in NSCLC cells, providing the foundation for future clinical translation with HDACis and Chk1 inhibitors that are currently in clinical studies for anticancer therapy. We expect that validation of Chk1 and its downstream effectors involved in G_2_/M checkpoint control as clinically relevant biomarkers of treatment efficacy could provide tools applicable to the enrichment of clinical trials and, hopefully, individualized tailoring of therapy of NSCLC.

## Materials and Methods

### Cell culture and experimental treatments

A549, H1299, H358, H441, H292, HCC827 (ATCC, Manassas, VA) and PC9 cells (provided by Dr. Matt Lazzara, University of Pennsylvania, Philadelphia, PA) were grown in RPMI supplemented with 5% fetal calf serum (FCS), 1% (v/v) penicillin-streptomycin, and 1% (v/v) L-glutamine at 37°C in a 5% CO_2_ incubator. Stock solutions of the Chk1 inhibitors UCN-01 (EMD Biosciences, San Diego, CA) and AZD7762 (Selleck Chemical), HDACis scriptaid (BioMol International, Plymouth Meeting, PA), LBH589 (Novartis, Basel, Switzerland), apicidin (EMD Biosciences, San Diego, CA) and MS-275 (Selleck Chemical) were dissolved in DMSO and added to the media at the indicated concentrations. Stock solution of the HDACi valproic acid (EMD Biosciences) was dissolved in water and added to the media at the indicated concentrations. Control cells were treated with vehicle alone.

### *Ex vivo* assays of primary NSCLC samples

Tumor cells were collected by fine needle aspiration technique, as previously described [39], from surgically resected Moffitt Cancer Center patient tumors. The tissue studies were approved by our Institutional Review Board at the University of South Florida. Briefly, tumor cells were obtained with sterile 23-gauge needles, and all samples were confirmed to be enriched for cancer cells by microscopic assessment. Approximately 25,000 tumor cells were treated in duplicate with vehicle (control) or LBH589 (40 nM) in a humidified 5% CO_2_ incubator at 37°C for 18 hours. No fibroblast or endothelial cell growth was observed. Following treatment, non-adherent and adherent cells (only a few, collected by scraping) were pooled together and centrifuged at 500×*g* for 5 minutes at 4°C. After they were washed with PBS, cells were lysed in 100 µL of ice-cold lysis buffer [50 mmol/L Tris-HCl, 0.25 mol/L NaCl, 0.1% (v/v) Triton X-100, 1 mmol/L EDTA, 50 mmol/L NaF, and 0.1 mmol/L Na_3_VO_4_ (pH 7.4)] containing protease and phosphatase inhibitors (Roche Molecular Biochemicals, Indianapolis, IN) and analyzed by Western blot.

### Antibodies

Mouse total Chk1, mouse total Chk2, rabbit cdc2 Y15P, rabbit cdc25c S216P, rabbit acetylated histones H3 and H4 (acetyl-H3 or acetyl-H4), rabbit total cdc2, rabbit total cdc25c, rabbit total cdc25a, rabbit total histone H3, rabbit cPARP, rabbit total AKT, rabbit H3 S10P, and rabbit total c-RAF antibodies were obtained from Cell Signaling Technology (Watertown, MA). Rabbit cyclin B1 antibody was obtained from AbCam (Cambridge, MA).

### Western blot analysis

Both adherent and detached cells in tissue culture wells were collected in 15-mL conical tubes and centrifuged at 4°C for 5 minutes at 1000 rpm in an Eppendorf 5810R centrifuge. The supernatant was removed, and the cell pellet was rinsed with ice-cold TBS, after which RIPA lysis buffer was added. Samples were sonicated, vortexed on ice every 10 minutes for 30 minutes, and then transferred to 1.5-mL microcentrifuge tubes and centrifuged for 10 minutes at 13,000 rpm at 4°C in an Eppendorf 5417R microcentrifuge. We used the Pierce BCA assay kit to determine protein concentrations, following manufacturer's protocol (Thermo Fisher Scientific, Rockford, IL). Samples were heated to 95°C for 10 minutes prior to resolving on an SDS-PAGE using a 4% stacking/10% resolving gel and transferred to a polyvinylidene difluoride membrane using a semi-dry transfer device (BioRad Industries, Hercules, CA). The membrane was blocked for 1 hour at room temperature in Pierce Superblock (Thermo Fisher Scientific) and probed for various antibodies. Enhanced chemiluminescent detection was performed following manufacturer's protocols (Thermo Fisher Scientific). Quantitation of Western Blots was done using AlphaEase FC software (Cell Biosciences, Inc., Santa Clara, CA).

### Determination of Annexin V Positive Cells by Flow Cytometry

A549 or PC9 NSCLC cells were seeded in 6-well plates at a density of (4×10^5^) cells/well. Cells were treated the next day with 40 nM LBH589 and collected 24 hours later for analysis using the BD Annexin V-FITC/7-AAD Flow Cytometry kit (BD Bioscience, San Jose, CA, 559763) per manufacturer's protocol. Cells were trypsinized with 0.05% w/v trypsin (Invitrogen Corp, Carlsbad, CA, 25300) and centrifuged for 5 minutes at 1000 rpm at 4°C in an Eppendorf Model 5417R centrifuge. Cell pellets were then rinsed 1x with ice-cold 1x DPBS and centrifuged again for 5 minutes at 1000 rpm and 4°C. Cells were then re-suspended in 1x Annexin V Binding Buffer at a concentration of 1×10^6^ cells/mL. An aliquot of 100 uL of this cell suspension was then stained by addition of 5 uL Annexin V-FITC solution and 10 uL 7-AAD solution and allowed to incubate for 15 minutes on ice in the dark. Positive control cells were prepared by heating an aliquot of cells to 98°C for 2 minutes. Separate aliquots of cells were prepared for Annexin V-FITC only controls and 7-AAD only controls. Aliquots of healthy, untreated cells were added to these controls post-heating to obtain a representative profile of healthy and unhealthy populations for gating. After the 15 minute incubation was completed, 400 uL of Annexin V Binding Buffer was added to each sample and mixed. Samples were analyzed within 30 minutes on a BD FACScan instrument with FLOWJO software to determine the percentage of Annexin V positive cells.

### Transfection experiments

The pGFP-CHK1 plasmid was purchased from Dana Farber/Harvard Cancer Center DNA Resource Core. The pFLAG-CHK1 plasmid was provided by Dr. Helen M. Piwnica-Worms, Washington University, St. Louis, MO. All transfections were carried out in 6-well culture plates maintained at 37°C in a 5% CO_2_ incubator. Twenty-four hours before transfection, 5×10^5^ A549 cells were seeded in each well. Cells were transfected with 1 µg plasmid DNA using Lipofectamine with Plus Reagent (Invitrogen, Carlsbad, CA) according to the manufacturer's protocols. Twenty-four hours post-transfection, cells were treated with HDACi for 24 hours prior to collection for Western blot analysis. Untransfected control cells were collected 48 hours post-transfection.

### Cell growth and viability assays

A549 cells were treated with LBH589 and UCN-01 alone or in combination at a constant ratio for 72 hours. Cell viability was measured by the CT-Blue assay (Promega). The combined effects of LBH589 and UCN-O1 were quantified using a combination index (CI) method developed by Chou and Talalay [40]. This method involves plotting dose-effect curves for each agent and their combination, using a median-effect equation: fa/fu  =  (D/Dm)*m*, where D is dose of drug, Dm is dose required for a 50% effect (equivalent to IC_50_), fa and fu are affected and unaffected fractions, respectively (fa  = 1 - fu), and *m* is the exponent signifying the sigmoidicity of the dose-effect curve. The computer software Xlfit version 4.3.1 (ID Business Solutions) was used to calculate the values of Dm and *m*. The CI used for the analysis of the drug combinations was determined by the isobologram equation for mutually nonexclusive drugs that have different modes of action: CI  = (D)1/(D*x*)1+ (D)2/(D*x*)2+ [(D)1(D)2]/[(D*x*)1(D*x*)2], where (D)1 and (D)2 are relative concentrations of drugs 1 and 2 and *x* is the percentage of inhibition. CI <1, CI  = 1, and CI >1 indicate synergism, additive effects, and antagonism, respectively.

### Cell cycle analysis

After they were rinsed with PBS, A549 or PC9 cells were collected at various time points after HDACi treatment and fixed in 70% ethanol while vortexing. Fixed cells were stored overnight at −20°C. The following day, cells were washed twice in PBS and treated with RNase A at 37°C for 30 minutes. Propidium iodide was then added to the cells, and cells were allowed to incubate in the dark for 1 hour at room temperature. Cells were then analyzed by flow cytometry using a fluorescence-activated cell sorter (BD Bioscience, San Jose, CA), Cell-Quest and FlowJo analysis software.

### Immunocytochemistry

A549 cells were grown to 50% confluence on sterile glass coverslips in 6-well plates. Twenty-four hours after they were seeded, cells were treated with 40 nM LBH589 for 24 hours. Cells were then fixed in ice-cold 4% (w/v) p-formaldehyde in PBS for 15 minutes and permeabilized with 0.15% Triton X-100 for 30 minutes. Cells were then blocked with 5% bovine serum albumin (Thermo Fisher Scientific) in PBS for 1 hour at room temperature and probed with rabbit cPARP antibody (Cell Signaling Technology) at a dilution of 1∶50 overnight at 4°C. After they were washed three times in PBS, cells were incubated for 1 hour at room temperature with AlexaFluor 596 conjugated goat anti-rabbit secondary antibody (Molecular Probes, Eugene, OR) at a dilution of 1∶200. Coverslips were then mounted on slides using Vector Mount containing DAPI nuclear stain (Molecular Probes). We viewed slides with a Zeiss-inverted microscope, analyzed with Axiovert software.

### Quantitative reverse transcription-PCR

RNA was isolated using the RNeasy kit (Qiagen 74104). First strand cDNA was synthesized from 1 µg of RNA using Bio-Rad iScript cDNA synthesis kit (Bio-Rad, 170-8891) in 20 ul reaction volume. Real-time PCR was performed using Bio-Rad iQ SYBR Green Supermix (Bio-Rad, 170-8882) on a MyiQ Single Color Real-time PCR detection system. 1 ul of cDNA was used in each 25 ul PCR reaction. The following primers were used: CHK1 forward, 5′-TGC TCA GAG ATT CTT CCA TC-3′; CHK1 reverse, 5′-AAC GCT CAC GAT TAT TAT ACC-3′; GAPDH forward, 5′-GAG TCA ACG GAT TTG GTC GT-3′; GAPDH reverse, 5′-TTGATTTTGGAGGGATCTCG-3′. GAPDH was served as internal control.
